# The Prevention of Ophthalmia of the New Born

**Published:** 1881-12

**Authors:** 


### ORIGINAL TRANSLATIONS AND ABSTRACTS.

### THE PREVENTION OF OPHTHALMIA OF THE NEW BORN.

Prof. Crede, of Leipzig, in a recent article (*Archiv fuer Gynækologie*, XVII, 1.), makes the following remarks about this sometimes very troublesome affection.

Ophthalmia neonatorum occurs rarely among the well-to-do, is more frequent among the working classes, but is an ever present evil in lying-in asylums.

Most obstetricians will probably agree with me in the opinion that the frequent inflammations and catarrhs of the vagina depend upon gonorrhœal infection, and that the contagious character of the secretions persists long after the specific gonorrhœal appearances are no longer evident. It may even be said that in cases where there is barely any secretion remaining, the contagion has taken place in the vagina if a conjunctivitis is developed within a few days after the birth of the child.

I am convinced from my observations that, almost without exception, all children who become affected with ophthalmia in the Leipzig Lying-in Asylum, become infected by the vaginal secretion during the parturient act. The disease begins as a rule two or three days after birth, but may begin earlier or later; the sooner it comes on the more intense is generally the inflammation.

In order to prevent the extensive ravages of the disease, I first tried the thorough and appropriate cleansing and treatment of the diseased mucous membrane of the vagina of the pregnant and parturient mothers. The results were, however, not satisfactory; the number of cases of ophthalmia became less, but the disease did not disappear altogether. I then began the direct treatment (disinfection) of the eyes of the children, and from that time the results were surprisingly favorable. I proceeded as follows: In all cases of women affected with gonorrhœa or chronic vaginal catarrh, admitted into the institution, the vagina was frequently washed out with warm water or a weak solution of carbolic or salicylic acid (2 per cent.). During labor the injections were made every half hour. The cases of ophthalmia became less in number but some cases were still obstinate and malignant.

In October, 1879, I first began using instillations into the eyes of the new-born immediately after birth, and employed for the purpose a solution of borax, (1–60) because I believed this remedy to be the mildest I could use. This was done only in children of diseased mothers in whom the above mentioned injections of the vagina had been made; but this method was not followed by the expected success, and two months afterward, I began the use of solution of nitrate of silver (1–40) which was instilled into the eyes shortly after birth. Before the use of the nitrate of silver, the eyes were carefully cleansed with a two per cent. solution of salicylic acid. Children treated in this manner remained healthy, but other children of apparently healthy mothers, in whom no prophylactic measures were taken, still became affected. Since June 1, 1880, the eyes of all children born in the institution, were disinfected by the instillation of one drop of a two per cent. solution of nitrate of silver after first cleansing the eyes by means of pure water; after that, a two per cent. solution of salicylic acid was applied to the eyes for twenty-four hours by means of linen compresses. The frequent vaginal douches were entirely omitted. All of the children so treated remained free from the slightest conjunctivitis, although many of the mothers had abundant vaginal discharges and granular vaginitis. Only one child became affected by a slight ophthalmia on the sixth day; and it was afterwards discovered that in the pressure of work, the prophylactic instillation had been omitted in this case.

No disadvantage followed this treatment. There was frequently a slight hyperæmia and increased secretion of the conjunctiva during the first twenty-four hours; but these rapidly disappeared. It is possible that the use of a weaker solution of nitrate of silver, if found effective, will avoid even these slight irritant effects.

The method of procedure is very simple, easily carried out, without danger, and it appears entirely successful.

My series of observations are, of course, as yet, too small to permit any certain conclusions to be drawn; but it is large enough and striking enough to urge to further trial. I would place the greatest importance upon the disinfection of the eyes of the infants and not of the vaginæ of the mothers in order to attain the desired results. Whether the method I have adopted is the most certain and best, will probably be decided in future, but at present I have no reason to depart from it.

From the statistics appended to the paper of Prof. Crede, it appears that in the year 1878, before the use of any but ordinary prophylactic measures the percentage of infants born in the lying-in asylum who became affected with ophthalmia, was nine and eight-tenths. For the first five months of the year 1880, when only the eyes of children born of diseased mothers were treated, the percentage of cases was seven and six-tenths, while from June 1st to December 8, 1880, the percentage was only five-tenths of one per cent., or one case in two hundred children, and as above remarked, in this case the prophylactic instillation of nitrate of silver had been inadvertently omitted.

Following the paper of Crede, came one by Olshausen (*Centralblatt fuer Gynækologie*, 1881, No. 2,), who uses a two per cent. solution of carbolic acid for the same purpose. Abegg, in Dantzig, follows with a brief paper, (*Archiv fuer Gynækologie*, Band 17, No. 3,), in which he regards the use of the nitrate of silver or carbolic acid solutions as unadvisable by midwives, and states that in the last ten years he had used in the lying-in asylum thorough washing of the eyes with pure water immediately after birth. In the 2,266 births which had taken place in that time, only 66 or 3 per cent. had ophthalmia.

